# Pulmonary Vascular Platform Models the Effects of Flow and Pressure on Endothelial Dysfunction in *BMPR2* Associated Pulmonary Arterial Hypertension

**DOI:** 10.3390/ijms19092561

**Published:** 2018-08-29

**Authors:** Reid W. D’Amico, Shannon Faley, Ha-na Shim, Joanna R. Prosser, Vineet Agrawal, Leon M. Bellan, James D. West

**Affiliations:** 1 Department of Biomedical Engineering, Vanderbilt University, Nashville, TN 37232, USA; 2 Department of Mechanical Engineering, Vanderbilt University, Nashville, TN 37232, USA; shannon.faley@vanderbilt.edu; 3 Division of Allergy, Pulmonary, and Critical Care Medicine, Vanderbilt University Medical Center, Nashville, TN 37232, USA; ha-na.shim@vumc.org (H.-n.S.); joanna.johnson@vumc.org (J.R.P.); vineet.agrawal@vumc.org (V.A.)

**Keywords:** pulmonary arterial hypertension, endothelial dysfunction, disease modeling

## Abstract

Endothelial dysfunction is a known consequence of bone morphogenetic protein type II receptor (*BMPR2*) mutations seen in pulmonary arterial hypertension (PAH). However, standard 2D cell culture models fail to mimic the mechanical environment seen in the pulmonary vasculature. Hydrogels have emerged as promising platforms for 3D disease modeling due to their tunable physical and biochemical properties. In order to recreate the mechanical stimuli seen in the pulmonary vasculature, we have created a novel 3D hydrogel-based pulmonary vasculature model (“artificial arteriole”) that reproduces the pulsatile flow rates and pressures seen in the human lung. Using this platform, we studied both *Bmpr2*^R899X^ and WT endothelial cells to better understand how the addition of oscillatory flow and physiological pressure influenced gene expression, cell morphology, and cell permeability. The addition of oscillatory flow and pressure resulted in several gene expression changes in both WT and *Bmpr2*^R899X^ cells. However, for many pathways with relevance to PAH etiology, *Bmpr2*^R899X^ cells responded differently when compared to the WT cells. *Bmpr2*^R899X^ cells were also found not to elongate in the direction of flow, and instead remained stagnant in morphology despite mechanical stimuli. The increased permeability of the *Bmpr2*^R899X^ layer was successfully reproduced in our artificial arteriole, with the addition of flow and pressure not leading to significant changes in permeability. Our artificial arteriole is the first to model many mechanical properties seen in the lung. Its tunability enables several new opportunities to study the endothelium in pulmonary vascular disease with increased control over environmental parameters.

## 1. Introduction

Pulmonary arterial hypertension (PAH) is a disease characterized by the progressive occlusion of small pulmonary arteries through the proliferation of vascular cells. This leads to an increase in pulmonary vascular resistance, and eventually results in death from right heart failure [1,2,3,4,5,6,7]. The heritable form of PAH due to bone morphogenetic protein type II receptor (*BMPR2*) mutations leads to significant alterations in transcriptome-wide expressions in both human lung and cells cultured in flasks [8,9,10]. Specifically, studies have found that the deregulation of the Ras/Rho GTPase pathway plays a large role in the cytoskeletal defects seen in PAH [8,10,11,12,13,14]. The cytoskeletal defects and endothelial dysfunction are widely observed in PAH patients, and have been linked to *BMPR2*-associated disease [8]. While it is known that endothelial cells are sensitive and reactive to mechanical forces like shear [15,16], the intersection of *BMPR2*-associated PAH and mechanical properties of the pulmonary vasculature is still largely unknown.

Investigators have been interested in the effects of mechanical stimuli (e.g., pressure and flow) on the endothelium for decades. Surgical trials in animals in the 1960s showed that alleviating pressure and flow alone largely resolved pulmonary vascular remodeling [17,18,19,20,21]. However, this level of fine control of local vascular mechanics is impractical when studying vascular disease in humans and is incapable of revealing important molecular consequences of pulmonary vascular remodeling. More recently, there has been a growing interest in examining some of these mechanical factors in the context of pulmonary hypertension, with recent papers investigating the effects of stiffness, flow, and stretch on pulmonary vascular cells [22,23,24]. There is substantial evidence that changes in the mechanical properties of the pulmonary vasculature are the critical determinants of disease progression. These mechanical stimuli override the effects of initiating events and result in common molecular and physiological pathology by the time the disease has become clinically relevant, regardless of initial etiology [25,26,27,28].

The aberrant gene expression and cytoskeletal architecture in *BMPR2* mutant pulmonary endothelial cells may contribute to endothelial dysfunction [8]. This endothelial dysfunction can manifest in a number of ways, including abnormal barrier function, deregulated cytoskeletal assembly, and improper cytoskeletal organization. However, all in vitro studies have examined cells in stagnant dishes and no studies have probed the effect of mechanical stimuli on driving the endothelial dysfunction in PAH. To date, it has not been possible to study the effects of pressure and flow in a relevant 3D model, as none of the earlier platforms used were capable of independently controlling these critical parameters.

To overcome this limitation, we have created a new artificial arteriole cell culture platform that comprises a hydrogel-based extracellular matrix, controlled perfusion system, and fluidic network. Pressures, shear stresses, and pulsatile flow are controlled by a microcontroller-operated system of pumps and channels, and each parameter may be set to a biologically-relevant value. Thus, we are able to mimic, in a 3D cell culture platform, the mechanical stimuli that are seen in the vessels in the lung.

This artificial arteriole platform presents an opportunity to study how oscillatory flow leads to differences in gene expression, morphology, and barrier function in *Bmpr2*^R899X^ and wild type (WT) murine pulmonary microvascular endothelial cells (MPMVECs) and thus provides a more biomimetic environment to study PAH in vitro.

## 2. Results

### 2.1. Engineering an Artificial Arteriole

A peristaltic pump and a microcontroller circuit were designed to enable pulsatile flow and tunable mechanical parameters within the model. The circuit was situated outside an incubator and was designed to control the peristaltic pump from within the incubator. The peristaltic pump was engineered to perfuse an artificial endothelium within a closed dish.

**Figure 1:**
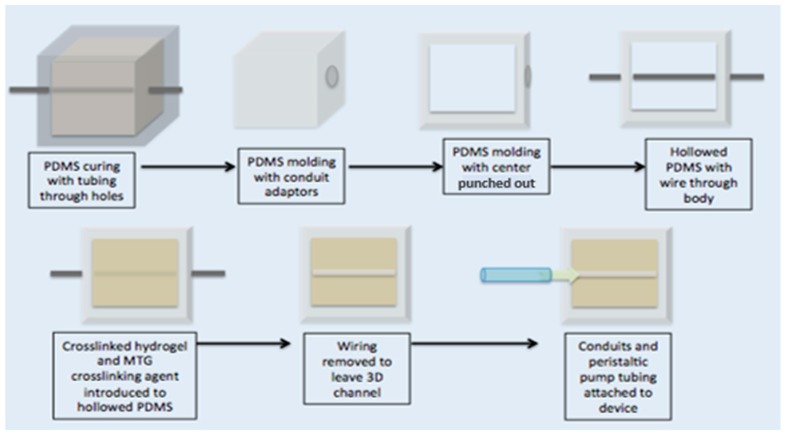
Schematic of artificial arteriole channel assembly. The artificial arteriole channel and PDMS mold construct was created through a series of molding and casting steps depicted above. The top row illustrates how to manufacture the PDMS mold and the bottom steps illustrate how to create the artificial arteriole construct.

The artificial endothelium channel was made of 10% gelatin crosslinked with 10% MTG in a 10:1 ratio. Young’s modulus was measured to be 131.89 Pa ± 13.07 Pa by an Instron 5944 (Norwood, MA, USA) equipped with 10 kN compression. A schematic depicting the manufacturing of the artificial endothelium channel can be seen in Figure 1.

By manipulating the vessel diameter, and conduit tubing diameter and length, we were able to change both the shear stresses and pressure waveforms within the artificial endothelium channel. The inner-channel pressure was successfully characterized within the center of the channel by a catheter inserted prior to each flow experiment.

While it was possible to generate several different pressure waveforms in the channel using our perfusion system, the waveform that was similar to the pressures in human pulmonary arterioles was chosen for experimentation. Devices designed to replicate the pulmonary arterioles generated stable pressure values, with a standard deviation of 0.68 mmHg. The platform chosen had a channel diameter of 900 µm and perfusion conduit tubing with a diameter of 1.5 mm.

**Figure 2:**
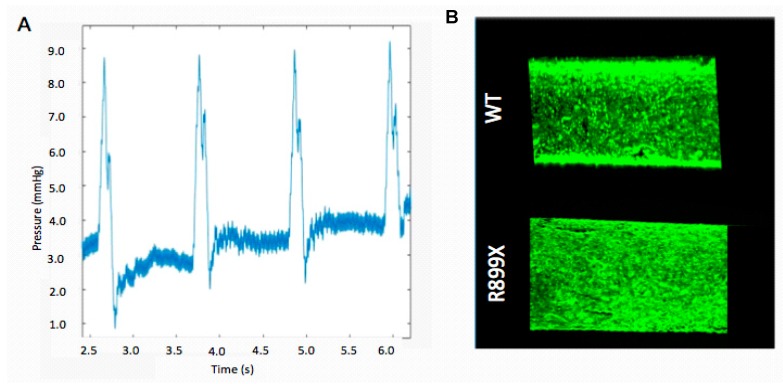
Validation of mechanical and cellular properties. (**A**) Sample waveform depicting the oscillatory pressure waves observed within the center of the channel. (**B**) WT and mutant *Bmpr2*^R899X^ endothelial cell channels imaged at 5× with fluorescent calcein AM to observe the monolayer formed by the cells. Channels were imaged after perfusion to ensure that the endothelial monolayer remained intact after the flow experiments. Cells were removed from conditional immortalization and the transgene was activated though doxycycline.

The fluidic resistance provided by this channel geometry facilitated the production of an oscillatory pressure wave with a pressure difference of about 9 mmHg that was sustained throughout the course of each experiment (Figure 2). Poiseuille’s law was used to estimate a shear stress of 98 dyn/cm^2^. During this flow pulse, the maximum flow rate within the channels was 800 µL/s and the minimum flow rate was 0 µL/s. Our chosen parameters for pressure and flow closely represented the data from initial physiological studies. Previous studies reported arteriole pressures of about 11 mmHg [29]. Flow rates for the pulmonary vasculature vary tremendously due to physiological variation in the diameters of the arterioles and capillaries. We chose 800 µL/s because it is within other reported flow rates of 0.6 µL/s and 50,000 µL/s [30,31].

### 2.2. Artificial Pulmonary Arteriole Validation.

Endothelium were formed by seeding the artificial arteriole with a total of 150,000 cells. After seeding, the cells lining the channels were allowed to proliferate until confluency. Channels were then perfused at an oscillatory flow rate of 800 µL/s with a pressure of 9 mmHg for one second, followed by one second of relaxation (pump off). Seeded channels were imaged to verify that an artificial endothelium remained following 24 h of oscillatory flow (Figure 2). Only devices without any notable acellular patches or defects were used for additional experimentation.

### 2.3. Oscillatory Flow Drives Genome-Wide Changes in WT and Bmpr2^R899X^ Pulmonary Endothelial Cells

The molecular consequences of oscillatory flow on the *Bmpr2*^R899X^ mutation were determined by performing RNA-Seq on an Illumina HiSeq system and measuring gene expression of both adult WT and *Bmpr2*^R899X^ MPMVECs that were control immortalized with transgenes activated. After exposure to flow, the changes in gene expression within the mutant and WT cells as well as between perfusion conditions were dramatic, with 457 genes changed more than 0.4 log times the un-perfused condition.

**Figure 3:**
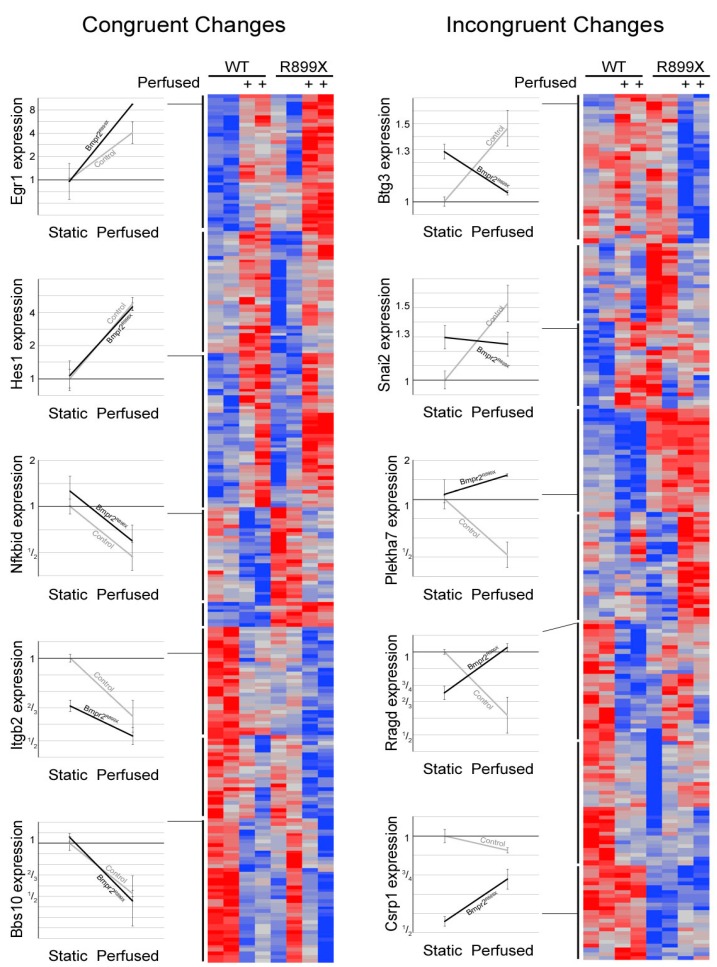
Congruent and incongruent heat map depiction of gene expression changes. Heat maps were separated by cell genotype (WT and *Bmpr2*^R899X^) with flow (+) or without flow. Representative genes and corresponding expression changes were selected to illustrate change in expression seen within the congruent and incongruent maps. Congruent genes had similar directional changes to flow but were offset by the magnitude of expression. Incongruent genes had opposite directional responses to flow that result in different magnitude changes. The inclusion of flow led to notable gene expression changes in both the R899X and WT endothelial cell. Notable changes in gene expression were seen between the cell’s genotype and flow conditions.

**Figure 4:**
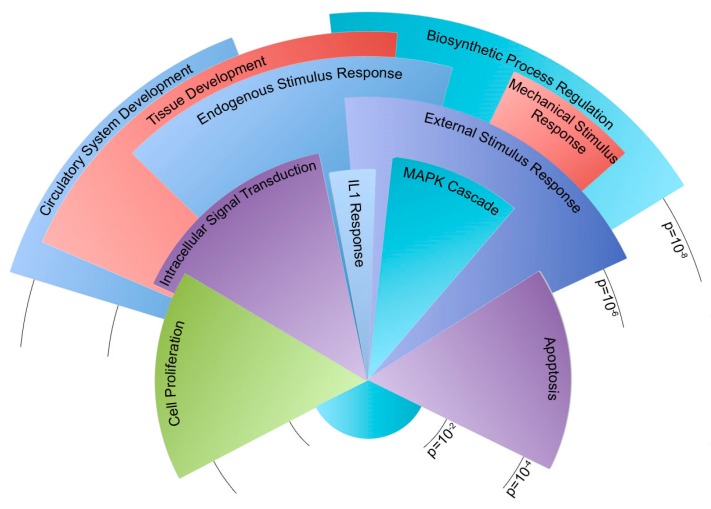
Congruent genes dysregulated in *Bmpr2*^R899X^ endothelial cells after 24 h of perfusion. Pie chart shows relative number of genes in statistically dysregulated gene ontology groups. Arc length of the gene ontology groups corresponds to the number of genes present in each group. Radius length corresponds to significance, where the longest categories were significantly dysregulated at ~ *p* < 1 × 10^−8^ and the shortest categories were dysregulated at *p* < 1 × 10^−3^. Please see the Supplementary Materials for a table of these findings.

Gene outcomes after exposure to flow were sorted into either a congruent or incongruent category (Figure 3). Congruent genes demonstrated similar directional changes in expression when exposed to flow, but are offset by different magnitudes in expression. A total of 248 genes were sorted into a congruent category. Of these congruent genes, 202 have functional annotation and 131 fell into statistically overrepresented gene ontology biological process groups. Figure 4 illustrates gene ontology groups by significance and number of genes.

**Figure 5:**
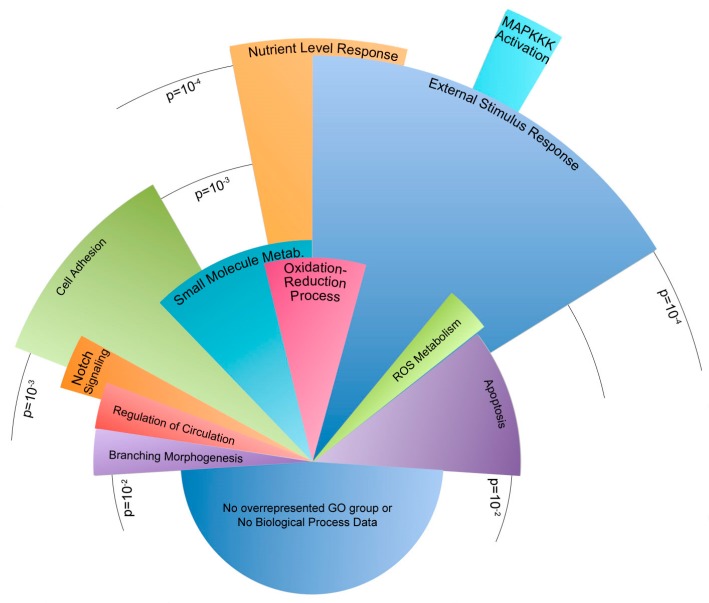
Incongruent genes dysregulated in *Bmpr2*^R899X^ endothelial cells after 24 h of perfusion. Pie chart shows relative number of genes in statistically dysregulated gene ontology groups. Arc length of the gene ontology groups corresponds to the number of genes present in each group. Radius length corresponds to significance, where the longest categories were significantly dysregulated at ~*p* < 1 × 10^−5^ and the shortest categories were dysregulated at *p* < 1 × 10^−2^. Please see the Supplementary Materials for a table of these findings.

The incongruent category is characterized by expression behavior that diverges when the cells are exposed to flow. For example, the introduction of flow may lead to an increase in expression in one group and a decrease in expression in another. A total of 209 genes were sorted into an incongruent category. Of these incongruent genes, 148 have functional annotation and 77 fell into statistically overrepresented gene ontology biological process groups (Figure 5).

Within the congruent category, the majority of genes were involved in the development of the circulatory system and other tissues. Other genes corresponded to the regulation of cell proliferation, cell death, and responses to external and mechanical stimuli. Similarly, many incongruent genes were involved in sensing external stimuli and the apoptotic process. Genes involved in cell adhesion were the most notably inconsistent gene ontology group when the congruent category was compared to the incongruent scenario. Both incongruent and congruent genes had ontologies correlating to mechanisms concerning response to external mechanical stimuli. In particular, the differences in cellular adhesion and cytoskeletal architecture were known to be different between *Bmpr2*^R899X^ and WT cells.

### 2.4. Bmpr2^R899X^ Pulmonary Endothelial Cells Do Not Change Morphology in Response to Flow

**Figure 6:**
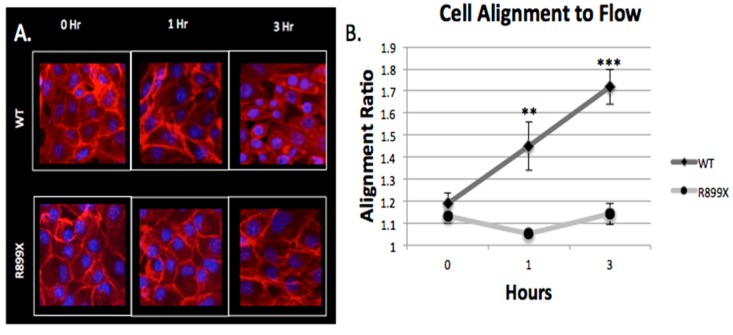
*Bmpr2*^R899X^ endothelial cells do not align in the direction of flow. (**A**) Morphological differences were seen in the WT cells as the amount of perfusion time increases, whereas the *Bmpr2*^R899X^ cells did not show evidence of elongation. Images were taken at 20×. (**B**) Without perfusion, both *Bmpr2*^R899X^ and WT endothelial cells showed no difference in cell morphology and alignment ratio (*p* > 0.05). After 1 h of perfusion, the WT cells started to align, but the *Bmpr2*^R899X^ cells showed no evidence of aligning in the direction of flow (*p* < 1.0 × 10^−4^). The WT cells continued to elongate and increase their alignment ratio while the *Bmpr2*^R899X^ cells maintained a nearly constant alignment ratio (*p* < 1.0 × 10^−5^). *N* = 3 for all cells (*Bmpr2*^R899X^ and WT) in all time points.

Endothelial monolayers were imaged at three different time points (*t* = 0,1, and 3 h) to assess morphological response to flow within the artificial arteriole. As seen before [32,33,34], WT cells respond to flow by slowly elongating in the direction of shear. Conversely, the mutant *Bmpr2*^R899X^ did not respond to flow and did not elongate after 3 h of exposure to perfusion (Figure 6).

This difference in alignment between *Bmpr2*^R899X^ and WT was drastic after 3 h of perfusion (*p* < 1.0 × 10^−5^). WT cells responded to flow within an hour while the mutant cells showed no sign of elongation at 1 h (*p* < 1.0 × 10^−4^). Before perfusion, both the WT and *Bmpr2*^R899X^ cells had nearly indistinguishable cell morphologies (*p* > 0.05), with a similar alignment ratio of about 1.1 (measured by the ratio of cell’s length in the flow direction divided by the length perpendicular to flow). The difference in morphology highlights the role that cellular adhesion and cytoskeletal genes may play in the mutant and WT cells when exposed to flow.

### 2.5. Bmpr2^R899X^ Pulmonary Endothelial Cells Demonstrate Significant Barrier Dysfunction In-Vitro

**Figure 7:**
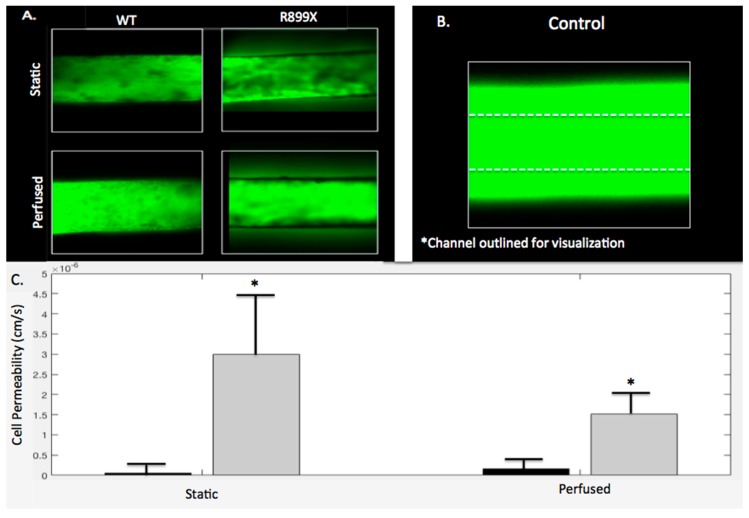
*Bmpr2*^R899X^ MPMVECs have increased permeability compared to WT cells under both perfusion and static conditions. (**A**) Static and perfused WT and *Bmpr2*^R899X^ channels were imaged at 5× magnification following one hour perfusion with FITC dextran. The channel diameter is 900 µm (**B**) A control channel without cells was perfused with FITC dextran for comparison. Because of excessive fluorescence due to high permeability, the outline of the channel was added. (**C**) Permeabilities of the WT and *Bmpr2*^R899X^ are plotted under both conditions to visualize magnitude differences in permeability between genotype. No statistical difference was seen between perfusion and static conditions in all genotypes (*p* > 0.05); however WT and *Bmpr2*^R899X^ channels had significantly different permeabilities under both static and perfusion conditions (*p* < 0.05). *N* = 3 for all cells (*Bmpr2*^R899X^ and WT) in all conditions (static and perfused).

Channels and gels were imaged following either perfusion (*t* = 3 h) or static conditions (*t* = 0 h) to assess the barrier function of the *Bmpr2*^R899X^ and WT endothelium lining the channels. The WT endothelial cells maintained barrier integrity when perfused with 10k FITC dextran in both perfused and static conditions with permeabilities of 1.65 × 10^−7^ and 5.93 × 10^−8^ cm/s, respectively. There was no significant difference between perfused and static WT channels (*p* > 0.05). As depicted in Figure 7A, little FITC dextran was seen leaking out of the WT channel and into the hydrogel. Unlike the WT experiments, the *Bmpr2*^R899X^ endothelialized channels exhibited more FITC dextran leakage in both the perfusion and static conditions, with permeabilities of 1.51 × 10^−6^ and 3.00 × 10^−6^ cm/s, respectively. This increased permeability can also be seen in Figure 7A. Similarly to the WT channels, *Bmpr2*^R899X^ channels had no significant differences in permeability in static or perfusion conditions (*p* > 0.05). A control channel without any endothelium had a greater amount of leakage than all seeded channels, regardless of the mutation. The control channel seen in Figure 7B has been outlined in order to see where it is located, as the fluorescence from the FITC dextran diffusion obfuscates the channel boundary. In both the perfusion and the static conditions, the WT channels were always less permeable than the *Bmpr2*^R899X^ channels, with *p* < 0.05 for both static and perfusion conditions (Figure 7C).

## 3. Discussion

Several groups have used microfluidic systems to model vascular shear to study the responses of endothelial cells to mechanical stimuli [35,36,37,38]. Inspired by these early characterizations of endothelial cells in the presence flow, we developed an artificial arteriole to study pulmonary vascular disease. When designing our platform, we sought to incorporate tunable parameters like shear stress, pressure, oscillatory flow, and crosslinking-controlled matrix stiffness so that a wider range of biological questions could be asked.

Before experimentation, all devices and platforms were tested for mechanical and cellular consistency prior to data collection (Figure 2). RNA-Seq from WT and *Bmpr2*^R899X^ MPMVECs cultured under static and perfusion conditions demonstrated that many central pathways relevant to PAH etiology were altered in the mutant cells when exposed to flow (Figure 3). These findings were stratified into two categories: congruent or incongruent gene expression changes. The majority of significant genes discovered were in the congruent gene category. In this category, many of the gene pathways were involved in the development of the circulatory system, and may hint at the progression or penetrance of PAH (Figure 4). In most cases, the *Bmpr2*^R899X^ cells demonstrated a greater level of gene expression when compared to the WT cells. The *Bmpr2*^R899X^ cells tended to overrespond to many key molecular pathways responsible for endothelial maintenance and sensing, but these responses mirrored the up or down regulation patterns seen in the WT cells. The RNA Seq revealed that many of the pathways altered in response to pressure and flow may have relevance to *BMPR2*-associated PAH, and should be explored to further elucidate how mechanical stimuli may affect disease outcomes.

Gene expression in the incongruent category showed differing gene expression changes to many gene ontologies relevant to PAH. In many instances, the WT cells had up or down gene expression changes in response to flow, but the mutant *Bmpr2*^R899X^ cells would often have a contradictory response. The most notable response was found in genes that regulate the cytoskeleton and regulate how cells respond to external stimuli (Figure 5). The deregulated cytoskeleton pathways have been observed before in traditional cell culture and align with the findings in our data [8,39,40,41]. The induction of mechanical stress (e.g., oscillatory pressure and flow) further exacerbated an already deregulated cytoskeletal profile and informs us that the mechanical environment in PAH may help drive disease.

Evidence of an altered response to flow and pressure was observed in the *Bmpr2*^R899X^ cell’s morphology when the channels were first perfused (Figure 6). Endothelial cells are known to respond to flow [32,33,34], but the aberrant alignment of the *Bmpr2*^R899X^ cells may be correlated with the deregulated cytoskeletal and mechanical response pathways. The inability of the *Bmpr2*^R899X^ mutant cells to adapt to flow conditions may explain many of the endothelial defects known to take place in PAH [42,43,44]. To our knowledge, this is the first report that the *Bmpr2*^R899X^ endothelial cells do not align to flow, and may also be the first report of an endothelial cell that did not align in flow conditions.

Endothelial dysfunctional and permeability issues are a known hallmark of *Bmpr2*^R899X^ PAH [8]. Previous studies have observed that perfusion of Evan’s Blue through the pulmonary vasculature of *Bmpr2*^R899X^ mice results in observable leakage that is much higher than in control mice [8]. To recapitulate this behavior in our platform, we tested the permeability of *Bmpr2*^R899X^ and WT endothelial MPMVECs cultured in our channels. Channels either experienced no flow or were perfused for 3 h to replicate the longest time point seen in the alignment study. After cells were exposed to oscillatory flow and perfused with dextran, fluorescence intensity values were measured within the channel, and within the region dextran diffused into the hydrogel. Permeability values were calculated for the *Bmpr2*^R899X^ and WT cells in both perfused and static conditions. Figure 7 illustrates how the WT cells maintain a greater barrier to 10k dextran in both perfusion and static conditions when compared to the *Bmpr2*^R899X^ cells (*p* < 0.05). The *Bmpr2*^R899X^ cells are more permeable to both the WT cells in both the static and perfused condition, with the perfused condition not increasing the rate of permeability. The lack of significant differences in permeability under flow suggests that the pathologic barrier function in PAH may be independent of physiological pressure or flow.

The increased permeability of the *Bmpr2*^R899X^ cells has been previously observed in vivo, but this is the first time similar increased permeability (as well as lack of cellular alignment) has been demonstrated in a 3D hydrogel culture model. No studies have looked at the impact of PAH’s heritable *Bmpr2*^R899X^ mutation and its changes in flow environments. These findings demonstrate how mechanical parameters often neglected in 2D cell culture yield important information needed to understand the pathology of PAH.

Our artificial arteriole recapitulated previously discovered deregulated gene expression pathways seen in 2D cell culture, and also re-created the increased permeability of *Bmpr2*^R899X^ endothelium seen in mice. Our model advanced understanding on how flow and pressure within the pulmonary microvasculature may lead to several gene expression changes directly related to disease progression and development. The artificial arteriole also enabled the novel discovery that *Bmpr2*^R899X^ cells do not elongate in the direction of flow, hinting at the possible correlation between deregulated sensing or cytoskeletal pathways and cellular adaptation to mechanical stress.

Future studies should aim to learn how the artificial arteriole could be used to model various stages of PAH. Pressure severity can be manipulated by using outlet tubing to control fluidic resistance within the channel. We do not know how the endothelium changes as the disease worsens, and this model may enable us to elucidate genotypic and phenotypic consequences as PAH changes. It is also important to learn how other ECM compositions in the model may influence endothelial behavior. Additionally, co-culture with other relevant cell types could enable novel studies of cellular interactions in this disease model and lead to greater understanding of other disease phenomena like inflammation.

## 4. Materials and Methods

### 4.1. Device Construction

A transparent PDMS (Sylgard 184, Dow Corning, Midland, MI, USA, mixed at 1:10 ratio) compartment was fabricated to house the artificial pulmonary vessel. This transparent mold was 12.5 mm × 12.5 mm with a depth of 7 mm. Holes were cut in the PDMS mold approximately 2 mm above the surface so that the artificial vessel was close to the surface of the PDMS, thereby facilitating high resolution imaging. If the channel were too close to the PDMS mold, anisotropic mechanical effects from the surrounding PDMS or ECM environment could be significant [45]. The ideal height of the fabricated vessel should be about 500 µm from the surface of the mold [45]. A male luer lock to 1/16″ adapter was fitted to a hole on one end of the PDMS mold (Cole Parmer, Vernon Hills, IL, USA). A solution of 10% *w*/*v* porcine gelatin (Sigma Aldrich, St. Louis, MO, USA) was dissolved in EGM2 media containing doxycline (Lonza, Alpharetta, GA, USA). A crosslinking enzyme, microbial transglutaminase (MTG) (Modernist Pantry, Eliot, ME, USA) was dissolved in 10% *w*/*v* PBS and mixed at 10% *w*/*v* with the gelatin to induce chemical crosslinking [46]. A 900-µm diameter steel rod served as a template to define the diameter of the artificial vessel. The rod was threaded through the PDMS holes and male luer adapters, and the MTG and gelatin solution was poured into the PDMS mold at 37 °C. Devices were covered and placed in 37 °C incubator for 20 min. After gelation, the rod was pulled from one side of the gelatin until completely removed. The hollow channels were then coated with 100 µg/mL collagen IV (Sigma Aldrich, St. Louis, MO, USA) and 100 µg/mL fibronectin (Sigma Aldrich, St. Louis, MO, USA) for 48 h before cell seeding. During coating, devices are kept submerged in media. See schematic representation of fabrication process in the Supplementary Materials.

Poiseuille’s law was used to extract pressures at various lengths and radii from the channel geometry. Poiseuille’s law defines the relationship below:

(1) $$Q\;=\;\frac{{\Delta P\pi r\;}^{4}}{8L\eta }$$

After characterizing multiple diameter configurations, a 900-µm channel was selected for experimentation. The 900-µm channel had a shear stress of 98 dyn/cm^2^ and a shear rate of 11,178 s^−1^ The flow rate during a 100-ms pulse time was measured to be 800 µL/s.

A DC peristaltic pump (Adafruit Industries, New York, NY, USA) was used to mimic the pulsatile nature of the heart. An Arduino microcontroller (Arduino, Turin, Italy) was used to power and control the pump duty cycle. The Arduino code provided pulses at 1 Hz, or the standard human heart rate at 60 beats per minute. After the artificial arteriole was assembled, pressure waveforms were verified within the channel using a pressure sensitive catheter (Miller Instruments, Houston, TX, USA) at the center of the 900-µm channel.

### 4.2. Bmpr2^R899X^ and WT Cells

*Bmpr2*^R899X^ cells were derived from Immortomouse X Rosa26-rtTA2 X TetO7- *Bmpr2*^R899X^. The immortomouse contained a transgenic insertion of the SV40 large T antigen, tsA58, under the control of an interferon inducible promoter [47]. When the cells were grown at 33 °C and the interferon added, the transgene was activated and the cells are immortalized. When the cells are moved to 37 °C, the transgene became inert. The immortalized cells proliferate as though they were immortalized at 33 °C, but revert to a normal mortal phenotype when cultured at 37 °C. The *BMPR2* mutation was induced by the addition of doxycycline to the media. The Immorto-*BMPR2* mutant pulmonary endothelial cells were harvested from mice as previously described [48].

### 4.3. Cell Seeding

*Bmpr2*^R899X^ and WT cells were seeded at a density of 75,000 cells/device. The following day, the devices were seeded with another 75,000 cells/device. Cells were seeded directly into the channel by inserting a pipette tip into the luer lock. The devices were rotated every 30 min for two hours to encourage coating of the entire channel. All cell seeding took place in the immortalized condition (33 °C with appropriate media) with the *BMPR2* mutation activated via doxycycline. 24 h prior to experimentation, conditional immortalization was turned off by moving the cells to 37 °C and removing the interferon. Seeded channels were housed in wells while in the incubator. During the seeding process, the level of media in the wells was below the height of the channel to ensure all cells remained in the channel. After the cells were attached, the media level was increased to the height of the channel.

### 4.4. Platform Set-Up

Two perpendicular holes about 40mm apart were drilled in medium polystyrene round containers (Ted Pella, Redding, CA, USA). Luer adapter fittings with lock nuts were inserted into the two drilled holes and secured with nylon panel lock nuts (Cole Parmer, Vernon Hills, IL, USA). The peristaltic pump was connected to the external conduits of the luer adapter. Within the container, one of the luer adapters was fitted with a 1.92 mm diameter tube (VWR, Randor, PA, USA) approximately 35 mm long that sat in the container. The other luer adapter was fitted with the same tubing but with a female luer lock to 1/16″ adapter fitted to the end. Prior to experimentation, the perfusion setup was submerged in bleach for 24 h and then 100% ethanol for 48 h. Immediately before use, the containers were aspirated of liquid, dried, and rinsed with PBS. The peristaltic pump was used to perfuse 100 mL of 100% ethanol and then 100 mL of PBS immediately before use. The containers were filled with 20 mL of media containing doxycycline (without immortalized conditions) via perfusion using the peristaltic pump until no air was left in the tubing. Fully endothelialized channels were attached to the female luer adapter within the container. The containers and the channels were placed in an incubator (Hera Cell 150) and connected to the microcontroller and then perfusion was initiated. See Supplementary Materials for images.

### 4.5. Assessment of Artificial Lumen

24 h after the second seeding, channels were perfused and then assessed for confluent monolayers around the entire cylinder. Channels were stained with calcein AM stain (Life Technologies, Carlsbad, CA, USA). A Zeiss LSM 710 Confocal Microscope was used to determine whether the cells formed a confluent lining on the channels. Channels that were not confluent were disconnected from perfusion and monitored until cells had proliferated such that confluency was achieved.

### 4.6. RNA Sequencing

Channels were perfused for 24 h. Each array consisted of two channels per condition (*Bmpr2*^R899X^ and WT and perfused or static). RNA-Seq was performed on an Illumina HiSeq (San Diego, CA, USA) system with a directional mRNA library prep, SR-50, with 30 million reads. RNA aligning was performed with TopHat (Toronto, ON, Canada) to verify consensus genome sequence using ultra high-throughput short read aligner Bowtie2. Gene ontology analyses were performed with WebGestalt (Houston, TX, USA) as previously reported [49,50].

### 4.7. Endothelial Alignment

The channels were perfused with media for 0, 1, and 3 h. Cells were immediately fixed and permeabilized within the channel. Cells were stained with phalloidin and DAPI and imaged with a Zeiss LSM 710 Confocal Microscope. Three devices were imaged per condition and a minimum of three images were taken per device. Alignment was quantified in ImageJ (Bethesda, MD, USA) by measuring the cellular length in the direction of flow and the cellular width perpendicular to flow. A ratio of the length to the width was calculated to quantify the elongation of the cell in response to flow.

### 4.8. Barrier Function

The channels were perfused with media for 0 and 3 h. Cells were perfused (30 µL/min) for 1 h with FITC-dextran (5 mg/mL, M_w_ = 5,000, Invitrogen, Carlsbad, CA, USA) and imaged every thirty seconds. The fluorescence signal of the permeabilized FITC-dextran was quantified in ImageJ and Matlab (Natick, MA, USA) (see code in Supplementary Materials). Intensity values were manually extracted from ImageJ in order to compensate for unanticipated channel movement. The diffusion coefficient of dextran within the gelatin was calculated before experimentation [51,52,53,54,55].

### 4.9. Statistical Analysis

Statistics were performed using a one or two factor ANOVA (+/− *BMPR2* mutation and +/− perfusion) with a Fisher’s exact post hoc test for comparisons between groups. P-values less than 0.05 were considered significant, but *p* values *p* < 0.1 were still used in the RNA seq analysis. Statistics were performed in R Studio (Boston, MA, USA).

## Supplementary Materials

Supplementary materials can be found at http://www.mdpi.com/1422-0067/19/9/2561/s1.

## Abbreviations

*BMPR2*	Bone Morphogenetic Protein Type II Receptor
MPMVEC	Murine Pulmonary Microvascular Endothelial Cells
MTG	Microbial transglutaminase
PAH	Pulmonary Arterial Hypertension
WT	Wild type
