# Supplementary material for: Pulmonary Vascular Platform Models the Effects of Flow and Pressure on Endothelial Dysfunction in BMPR2 Associated Pulmonary Arterial Hypertension
Source: Int J Mol Sci. 2018 Aug 29;19(9):2561. doi: 10.3390/ijms19092561 (PMC6164056; doi:10.3390/ijms19092561)
Supplement: Supplementary file 1 [file ijms-19-02561-s001.pdf]

Supplement:

Supplement 1: Tables of the Statistically Overrepresented Gene Ontology Groups (Congruent and Incongruent are below):

Statistically Overrepresented Gene Ontology Groups (Incongruent)

Description	# Genes	P-Value
activation of MAPKKK activity	3	1.40E-05
response to nutrient levels	10	5.09E-05
response to external stimulus	24	8.87E-05
single organism cell adhesion	12	0.000675186
Notch signaling pathway	5	0.002555792
small molecule metabolic process	18	0.006611771
regulation of blood circulation	5	0.006698247
reactive oxygen species metabolic process	5	0.007099366
morphogenesis of a branching structure	5	0.007804783
apoptotic process	17	0.008529415
oxidation-reduction process	12	0.009155532

Statistically Overrepresented Gene Ontology Groups (Congruent)

Description	# Genes	P-Value
circulatory system development	29	2.52E-09
negative regulation of biosynthetic process	36	2.75E-09
tissue development	40	6.47E-09
response to mechanical stimulus	13	1.36E-08
response to endogenous stimulus	34	2.67E-08
response to external stimulus	39	2.59E-07
regulation of intracellular signal transduction	30	3.93E-06
MAPK cascade	19	5.44E-06
cellular response to interleukin-1	7	1.16E-05
cell proliferation	33	1.48E-05
cell death	33	2.14E-05

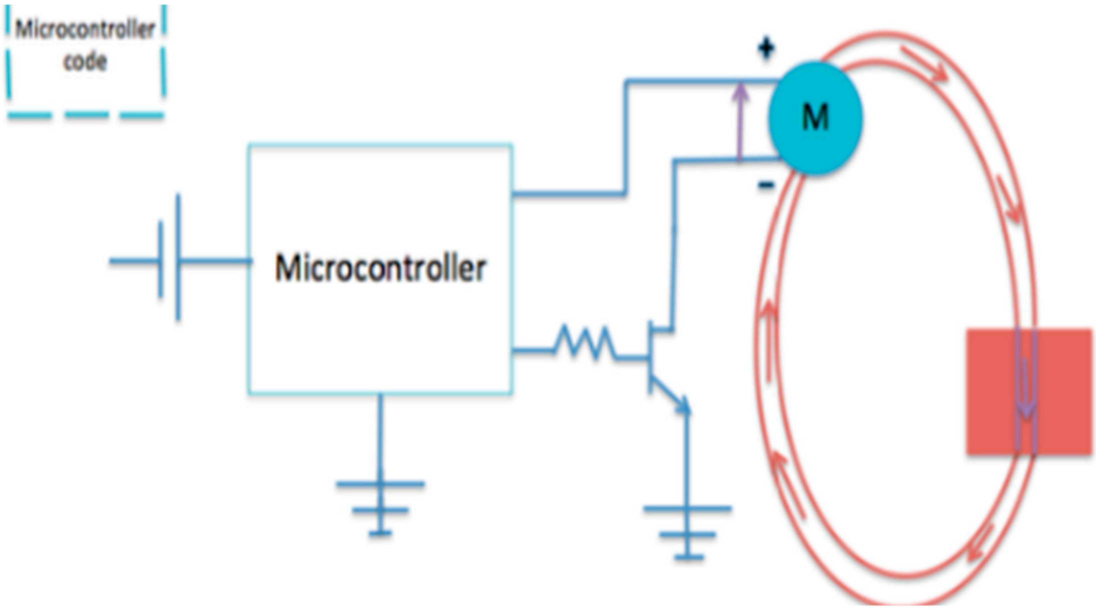

Supplement 2: Overview schematic of platform circuit and media circulation.

In **Supplement 3** an annotation of the PDMS mold and hydrogel is depicted. Below in **Supplement 4,** an image of the petri dish platform that houses the seeded and perfused devices is depicted.

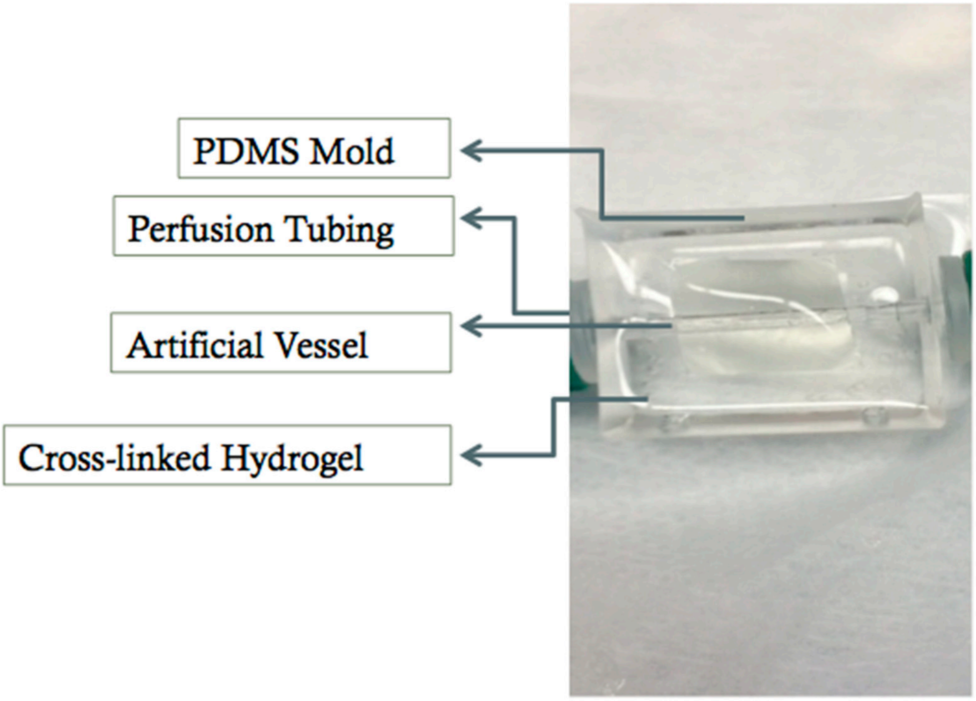

Supplement 3: Description of parts of the PDMS mold and hydrogel construct.

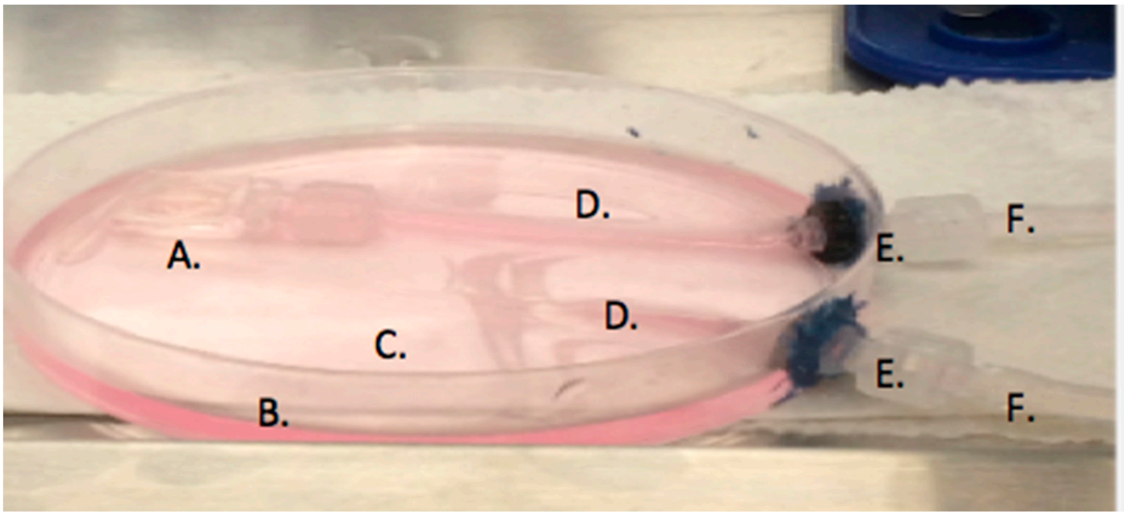

Supplement 4: Image of petri dish showing perfusion tubing, PDMS/hydrogel construct, and assembled platform. A) PDMS/Hydrogel device, B) Petri dish housing, C) Media reservoir, D) In/outflow tubing, E) Adapter conduits adhered to dish, F) Peristaltic pump perfusion tubing. Note: actual device perfusion and experimentation occurs within a 37C incubator

Below are the codes used to 1) program the Arduino microcontroller used for channel perfusion and regulation of oscillatory flow and 2) calculate permeabilities of cells from intensity values taken from ImageJ.

Arduino Code:

```
int motorPin = A1;
int blinkPin = 13;

int pumptime =100
; // how long pump is on
int waittime =1000; // how long pump is off

void setup() {
  // put your setup code here, to run once:
pinMode(motorPin, OUTPUT);
  pinMode(blinkPin, OUTPUT);
}

void loop() {
  // put your main code here, to run repeatedly:
digitalWrite(motorPin, HIGH);
  digitalWrite(blinkPin, HIGH);
  delay(pumptime);
  digitalWrite(motorPin, LOW);
  digitalWrite(blinkPin, LOW);
  delay(waittime);
}
```

Matlab Code:

```
%% Calculate permeability from confocal imaging datasets

        Dgelatin=20.9 %From FRAP Data 10k=12.6, 3k=20.9
        Distance_microns = 1120 %distance from channel edge to gel edge

        %%Import measurements from fiji macro
        Iedge = xlsread('C:\path\ GelBG.csv',1,'B2:B241');%%Average Intensity at no-flux region
        Channel_Io = xlsread('C:\pathChannelBG.csv',1,'B2:B241'); %%Intensity within channel
        Igel=  xlsread('C:\path\GelNearChannelBG.csv','B2:B241');%%Average intensity of gel

        %% Convert slice/frame number to time interval
        Slice = xlsread('C:\path\Profile.csv','Profile','C1:IH1');
        Time_minute = Slice*.5;
        Time_second = Time_minute*60;

        %%Calculating C for each timepoint
        C_numerator=Iedge-Channel_Io;
        C_denominator = Igel-Channel_Io;
        C=C_numerator./C_denominator;

        %% Graphing logC versus time
        x = Time_second;
        y = C;
        plot=semilogy(x,y);

        %%Fit logC versus time to extract Lambda (slope)
        curvefit = fitlm(x,y,'poly1');
                coeff=curvefit.Coefficients.Estimate;
                Rvalue=curvefit.Rsquared.Ordinary;
                output = xlswrite('C:\path\curvefit.xlsx', coeff,1);

        %Use Lambda to calculate K (permeability) in microns/second
        Lamda = xlsread('C:\path\curvefit.xlsx',1,'A2:A2');
        K=(Dgelatin/Distance_microns)*(Lamda*tan(Lamda));
        K_cm = K*10000; %% conversion to cm/s
                outputValue = {;'K cm/s';'Lambda';'Rsquared'}
                values=[;K_cm; K;Lamda;Rvalue];
                result= table(outputValue, values);
                %Save results in excel file
                writetable(result,'C:\path\Results.xlsx','Sheet',1,'Range','A1:B3')
```

Below are the tables of the genes used to generate the heat map.

Supplement Table 1: Congruent Genes used in the heat map

Gene Symbol	WT static	WT static 2	WT perfused	WT perfused 2	R899X static	R899X static 2	R899X perfused 2	R899X perfused
1010001N08Rik	1.5	1.5	1.3	0.8	2.0	1.9	1.5	1.1
1700123I01Rik	1.1	1.5	0.7	0.7	1.8	1.1	1.4	0.6
2610044O15Rik8	3.6	3.7	3.7	4.0	2.8	2.9	3.4	3.3
2900097C17Rik	1.1	1.4	1.9	2.3	3.4	3.8	4.3	3.6
3110001I22Rik	1.8	2.0	2.5	2.3	1.6	1.5	2.3	2.9
4930581F22Rik	0.3	0.8	0.2	-0.6	0.4	0.7	0.1	0.0
5830415F09Rik	3.1	3.2	4.1	3.7	2.4	2.9	3.6	3.4
9330179D12Rik	0.2	-0.6	0.3	0.3	-0.4	-0.2	0.5	0.2
A130014A01Rik	1.4	1.7	0.9	1.2	0.8	0.7	-0.1	-0.1
Adamts1	6.1	5.5	5.6	7.5	5.7	6.0	8.0	7.7
Ankrd1	5.4	5.9	6.6	7.0	5.1	5.2	6.3	7.7
Anks1b	1.1	0.6	-1.3	-0.6	1.2	0.8	0.1	0.6
Ankzf1	4.1	3.6	3.4	3.7	4.2	4.1	3.8	3.5
Apoe	0.2	-0.1	-0.4	-0.4	4.9	3.9	3.8	3.5
Arhgef4	0.8	0.9	-0.6	0.1	1.6	1.4	1.1	0.8
Arid5a	5.4	4.9	5.7	5.9	6.1	5.7	6.6	6.8
Armc5	3.8	4.0	4.2	4.2	3.7	4.2	4.3	4.6
Arrdc3	3.4	3.9	4.4	4.5	3.7	4.3	4.9	3.8
Atp5sl	4.2	4.1	3.7	3.7	3.9	3.8	3.6	3.8
Atxn7l2	2.5	2.3	2.9	3.1	2.4	2.4	2.9	2.5
Bbs10	2.2	2.0	1.1	1.2	2.2	2.3	1.5	0.5
Bhlhe40	7.0	7.2	8.2	8.2	6.8	6.8	8.2	7.5
Bora	4.6	4.6	5.0	5.3	4.9	4.6	5.1	5.2
Brd1	4.5	4.6	3.1	3.2	2.7	4.3	3.0	2.5
Btg2	5.2	5.0	6.7	6.5	6.6	4.5	8.8	8.3
C1qtnf6	3.5	3.5	2.3	2.8	2.8	3.7	3.3	2.1
Cacnb3	3.8	3.7	3.1	3.3	3.4	4.1	3.6	3.4
Ccdc64	3.9	3.7	3.4	3.3	3.5	3.6	2.9	3.0
Ccsap	3.5	3.5	2.9	2.7	3.5	3.2	2.8	2.9
Cdc34-ps	1.2	1.4	0.8	0.9	1.2	0.8	0.5	0.8

Cdkn2aip	5.6	5.0	5.8	6.1	5.3	5.2	5.8	5.6
Cenpv	3.8	4.1	3.6	3.5	3.4	3.8	3.2	3.4
Cep85l	0.1	-0.7	1.2	0.8	-0.5	-0.5	0.9	0.5
Ces2e	4.0	4.4	3.4	2.9	3.8	3.5	3.1	3.3
Cfap43	5.4	4.9	5.0	6.3	5.6	6.0	6.8	6.7
Chsy1	2.9	3.5	2.0	2.4	2.5	3.5	2.1	1.9
Cmtr2	4.7	4.5	4.9	5.3	4.5	4.8	5.4	5.4
Commd5	4.3	4.5	4.3	3.9	4.5	4.3	3.6	3.9
Coq10b	5.5	5.7	6.2	6.0	6.1	5.5	6.2	6.4
Cpz	0.7	0.6	0.0	-0.4	2.3	2.4	2.2	1.8
Crabp2	1.1	1.3	0.8	0.7	-0.2	-0.1	-1.2	-0.5
Csn3	1.1	1.3	0.4	0.5	-0.2	0.3	-0.5	-1.2
Csnk2a2	4.4	4.6	2.7	2.8	2.4	4.0	2.4	1.6
Cspp1	2.7	2.7	3.1	3.2	2.9	2.7	3.3	2.9
Csrnp1	4.7	4.6	6.2	6.7	5.7	4.2	7.4	6.9
Cul3	6.4	6.4	4.5	4.8	4.1	6.5	4.7	4.4
Cxx1a	3.5	3.5	3.0	3.2	3.2	3.2	3.0	2.9
Cxx1b	3.6	3.9	3.3	3.3	3.1	3.5	3.0	3.1
Cyhr1	6.8	6.4	4.2	5.2	4.1	6.3	5.2	3.8
Cyr61	7.3	7.4	10.6	10.7	8.1	7.4	10.2	11.3
Ddit4l	4.1	3.9	3.5	3.5	3.8	3.4	3.4	3.4
Dhrs3	2.5	2.7	1.9	2.0	2.5	3.0	2.4	2.5
Dnajc18	5.4	5.1	4.8	4.9	4.4	4.7	4.5	4.2
Dnhd1	1.0	0.4	0.0	-0.1	0.7	0.7	0.5	0.3
Dok1	5.6	5.3	5.2	5.2	6.1	5.9	5.5	5.4
Dtwd2	2.3	2.4	2.9	2.7	1.8	2.0	2.2	2.1
Dus4l	3.6	3.5	4.0	4.1	3.2	3.6	4.0	3.6
Dusp5	3.4	3.7	4.9	6.3	5.5	4.5	6.9	6.9
Dusp6	5.5	5.9	6.8	7.5	7.0	7.4	8.3	8.4
E130102H24Rik	0.5	0.7	-0.3	-0.4	0.5	0.5	0.1	0.2
Eda2r	4.7	5.0	4.9	5.2	4.3	4.7	5.1	5.1
Edn1	2.1	2.8	4.4	3.6	2.7	3.1	4.0	3.2
Egln1	4.9	5.1	3.2	3.1	2.6	4.3	2.3	2.2
Egr1	6.3	6.1	7.8	8.7	6.9	5.4	9.4	9.5
Egr2	-2.0	-2.0	0.6	3.2	-2.0	-1.0	4.9	5.5
Eid2b	3.4	2.8	3.0	2.7	3.1	3.2	2.6	2.7
Epha2	5.6	6.2	5.8	6.9	5.4	6.0	6.9	6.5
Errfi1	4.9	4.8	5.5	6.5	6.3	5.9	8.5	8.5

Exog	4.4	4.0	3.9	4.0	4.4	4.5	4.0	4.0
F3	3.6	3.8	4.5	4.5	5.6	5.2	6.2	7.4
Fam110c	0.7	0.6	1.3	0.8	4.3	4.5	4.8	5.0
Fam120a	6.5	7.2	5.6	5.8	5.9	6.7	5.8	5.4
Fam98c	3.8	3.5	3.7	3.3	3.7	3.6	3.1	3.4
Fblim1	2.4	2.3	1.6	1.9	4.0	4.4	3.8	4.0
Fbxo30	5.7	5.5	5.7	6.2	5.9	5.9	6.4	6.6
Fbxo44	2.6	2.4	1.8	1.6	3.1	2.9	2.9	2.5
Filip1l	4.0	4.0	4.4	4.5	3.3	3.7	4.3	4.6
Flt3l	2.4	2.4	1.9	2.0	2.7	2.2	2.4	2.2
Fos	4.2	4.5	8.0	6.3	5.8	3.9	8.0	7.7
Fosb	-1.6	-1.1	2.0	2.1	-1.0	-2.0	4.0	2.3
G2e3	4.6	4.5	5.0	5.2	4.6	5.0	5.0	5.0
Gadd45g	4.6	4.9	7.4	6.3	3.8	3.7	5.5	6.7
Gid8	3.3	3.3	2.7	3.0	3.1	3.5	3.2	2.8
Git1	6.1	5.9	4.0	4.7	4.3	6.1	5.0	3.7
Glrb	3.5	3.2	3.2	3.1	1.3	1.3	0.6	0.9
Gm10254	5.1	5.1	5.3	5.6	5.0	5.1	5.6	5.6
Gm12565	1.9	1.7	1.6	1.2	1.3	1.3	0.9	0.9
Gm12715	7.3	8.2	8.2	8.3	7.7	7.7	8.3	8.7
Gm12966	5.3	5.4	5.9	5.4	5.0	5.2	5.5	5.7
Gm14295	2.1	2.3	2.9	2.9	2.4	2.8	2.5	3.1
Gm15270	-0.2	-0.4	0.7	0.3	-0.3	-0.8	1.0	1.4
Gm17501	1.3	1.2	2.3	2.0	-1.1	-0.6	-0.1	1.2
Gm20541	1.7	1.6	2.2	2.2	-0.4	0.8	2.0	1.7
Gm26648	2.0	1.9	1.2	0.9	1.4	1.3	1.6	0.4
Gm42547	1.2	0.9	-0.5	-1.0	0.0	1.4	0.0	-1.2
Gm43579	1.3	1.6	0.6	0.7	1.0	0.7	0.2	0.2
Gm6685	0.2	0.0	0.6	0.8	-0.8	0.0	1.1	0.8
Gm6710	2.5	2.7	3.3	3.2	1.2	2.2	2.8	2.5
Gm7436	1.2	0.7	0.9	0.1	0.6	0.4	-0.3	-0.2
Gm8185	2.2	2.4	2.4	1.7	2.3	2.0	1.7	1.6
Gm8261	0.2	0.1	0.2	0.7	-0.2	0.3	1.0	0.9
Gm8752	2.4	2.7	2.6	2.9	1.9	2.3	2.7	2.9
Gm9726	2.3	2.2	1.4	1.5	1.6	2.3	1.6	1.0
Gpr137	3.3	3.1	2.8	2.6	3.1	3.3	3.0	2.6
Gpsm3	2.2	1.7	1.3	1.3	2.3	2.4	2.2	1.8

Guca1a	1.5	1.0	1.1	0.5	1.5	1.1	0.6	0.5
H2-Q2	4.8	4.7	4.8	4.3	2.8	3.1	2.6	2.5
Hbegf	3.2	3.4	4.4	4.9	3.3	2.9	4.1	4.1
Heca	4.1	3.6	0.9	1.8	1.6	3.7	1.8	0.2
Hes1	3.2	3.8	5.9	5.6	4.0	3.1	5.8	5.5
Hilpda	4.8	4.8	6.2	5.5	6.0	4.7	6.1	5.8
Hint3	3.7	3.8	3.6	3.1	3.5	3.7	3.4	3.3
Hlx	0.6	1.1	0.3	0.7	1.5	1.8	0.8	0.9
Hoxa9	2.8	2.7	2.6	2.3	2.4	1.8	1.3	1.2
Hoxb2	3.3	3.3	2.9	2.6	4.1	3.0	1.9	2.4
Hyal1	2.8	3.0	1.9	2.4	2.0	2.4	1.6	1.6
Iba57	1.5	1.9	2.1	2.7	1.4	1.6	2.6	2.5
Id1	4.0	4.7	6.6	6.4	6.5	4.8	6.6	6.1
Id2	3.8	3.8	5.5	5.0	4.2	2.8	4.1	4.2
Id3	7.2	7.3	9.0	8.5	8.5	7.5	8.7	8.7
Ier2	6.0	5.9	7.2	7.5	6.7	5.5	7.9	8.2
Ier5	4.6	4.2	5.1	5.5	5.7	4.6	6.5	6.6
Ifrd1	7.8	7.6	8.1	8.2	8.2	7.7	8.6	8.4
Irak2	5.7	5.6	6.0	6.0	6.1	6.1	6.4	6.6
Itgb2	2.8	2.9	1.9	2.3	2.2	2.3	1.8	2.0
Itgb7	5.5	5.1	4.5	4.5	4.0	4.3	4.0	3.7
Jun	5.9	5.7	7.5	6.8	7.7	5.3	8.7	7.8
Junb	7.2	7.2	8.1	7.8	7.9	6.7	8.5	8.6
Klf10	6.1	5.9	7.2	7.8	6.8	6.3	7.8	7.3
Klf11	5.3	4.7	5.2	6.1	4.9	4.8	5.6	5.3
Klf13	5.8	5.8	5.0	5.4	5.2	5.6	5.5	4.6
Lmnb1	4.8	4.4	4.8	5.8	4.0	3.9	4.7	4.3
Lpar1	-0.4	0.0	1.8	1.9	1.0	2.1	2.2	2.4
Luc7l3	4.8	5.0	4.3	4.6	4.4	4.3	3.9	4.2
Lypd1	0.0	-0.1	-0.8	-1.1	2.9	1.6	0.9	0.7
Lysmd3	4.6	4.4	4.9	5.5	4.5	4.7	5.3	5.0
Mafk	6.6	6.5	6.7	7.2	6.3	6.2	7.1	7.1
Mapk3	6.7	6.5	6.2	6.3	6.3	6.6	6.4	6.0
Mars2	4.0	4.0	4.1	4.6	3.7	3.9	4.3	4.2
Mbd2	6.6	6.5	3.6	4.6	3.7	6.7	4.3	2.8
Mex3d	5.0	5.1	2.6	3.8	3.1	5.0	3.6	2.3
Mir99ahg	1.7	1.8	2.4	2.6	1.8	1.5	2.5	2.1
Mms22l	5.0	5.2	5.1	5.3	4.6	4.8	5.1	5.2
Mri1	5.7	5.6	5.6	5.3	6.0	5.9	5.5	5.6
Mta1	5.0	5.0	2.9	4.0	3.3	4.9	3.7	2.6

Mxd4	4.4	4.4	3.8	3.7	3.9	3.3	3.7	3.2
Myc	6.9	7.0	8.1	7.8	8.1	7.7	8.7	8.4
Nabp1	4.3	4.4	4.5	5.4	5.2	4.7	6.0	5.8
Nfil3	5.7	5.4	6.1	6.1	6.1	5.4	6.2	6.2
Nfix	5.5	5.7	4.0	4.1	4.0	5.0	4.3	3.4
Nfkb1	4.4	4.7	3.8	3.7	3.5	4.4	4.0	3.2
Nfkbid	1.9	1.6	0.3	0.9	2.4	1.7	0.6	1.3
Nop58	6.1	6.4	6.6	7.0	6.4	6.4	6.9	6.7
Nppb	1.4	2.2	3.4	4.0	0.1	0.2	2.4	3.0
Nr4a1	2.5	2.5	5.7	6.0	5.0	2.2	8.4	8.6
Nudt16	4.3	4.2	4.2	3.5	4.7	4.5	3.9	4.0
Nufip2	6.0	6.0	5.8	7.0	6.2	5.9	7.4	6.8
Osbp	5.8	5.6	3.5	4.3	3.5	5.7	4.6	2.9
Oxld1	2.2	2.3	1.5	1.6	2.7	2.4	1.8	1.7
Palm3	1.3	1.4	1.9	1.4	0.1	0.4	1.0	0.9
Pcdhb22	1.6	1.3	1.8	1.4	1.0	1.2	1.6	1.4
Pcdhga8	3.8	4.6	4.2	2.8	3.8	3.6	1.4	2.4
Pdgfa	3.8	4.0	3.6	3.2	3.5	3.4	2.7	2.9
Pgam1-ps1	0.5	0.1	0.9	0.9	0.1	-0.1	0.5	0.4
Pgpep1	4.8	4.8	4.5	4.2	4.2	4.4	4.0	3.9
Phc3	2.8	3.1	3.2	3.7	2.5	2.9	3.9	3.4
Phf13	5.8	5.5	4.7	4.9	5.6	5.7	5.1	5.3
Platr16	1.3	1.3	1.7	1.8	0.9	1.3	1.4	1.5
Plk2	7.3	7.1	8.6	8.4	8.4	7.8	9.3	9.5
Ppt2	4.3	4.1	3.6	3.8	4.1	3.9	3.5	3.4
Pramef8	5.4	5.1	5.7	5.5	5.2	5.2	5.6	5.6
Prps1l3	5.4	5.2	5.6	5.8	5.7	5.5	5.7	5.8
Ptbp2	3.4	3.7	4.2	4.0	2.5	3.5	3.7	3.6
Ptp4a3	3.9	4.1	3.4	3.0	4.7	4.2	3.7	4.4
Pus7l	2.4	2.5	2.9	3.2	2.3	3.1	3.1	3.1
Qk	6.5	6.6	4.9	5.1	4.8	6.7	4.9	4.2
Rab30	1.8	2.0	2.6	2.5	1.3	1.6	1.8	1.9
Ranbp9	4.8	5.1	3.3	3.5	3.2	4.8	3.3	3.2
Rasl11b	2.1	1.8	3.0	3.2	2.5	2.3	3.2	3.7
Rdh10	2.9	2.8	3.0	3.2	2.5	2.7	3.2	3.0
Rgs2	-0.2	-0.5	2.4	1.2	0.6	-1.0	4.7	3.4
Rnd3	7.4	7.1	7.9	8.0	7.8	8.1	8.6	8.6
Rnf113a1	3.0	2.9	2.6	2.6	2.9	2.7	2.0	2.1
Rnf126	5.4	5.4	4.8	5.1	5.0	5.4	5.0	4.6
Rnf145	5.5	5.4	4.2	4.6	4.7	5.4	4.3	5.1

Rom1	2.3	1.7	1.7	1.1	1.2	1.6	0.7	0.2
RP23-325D10.4	0.9	0.8	-0.3	0.3	0.5	0.6	0.3	0.0
Rps12-ps19	0.3	0.9	0.2	0.1	0.1	0.4	-0.4	-0.3
Rrnad1	3.7	3.3	2.9	2.9	3.0	3.1	3.0	2.7
Rundc3a	0.6	0.7	0.1	-0.5	0.1	0.2	-0.5	0.1
Rxra	4.7	4.7	3.2	3.2	3.6	5.2	3.7	3.2
Samd1	2.4	3.0	1.6	1.7	1.5	1.8	1.2	0.4
Scrn2	2.7	2.6	2.2	2.2	3.2	3.2	2.8	3.2
Sertad1	5.4	5.5	6.3	6.4	6.1	5.1	6.7	7.0
Sertad3	3.9	3.5	2.6	3.3	4.4	4.0	3.1	3.3
Shisa4	3.2	3.3	2.8	2.7	2.5	2.5	2.3	2.3
Slc25a25	4.9	4.5	5.4	5.8	4.7	4.6	6.5	6.6
Slc25a32	4.3	4.5	4.9	5.0	4.6	4.6	4.8	4.9
Slc33a1	4.8	5.0	5.2	5.2	4.5	5.1	5.2	5.3
Slc38a2	7.1	7.2	7.0	8.5	6.7	7.5	8.7	8.2
Slc4a11	3.7	3.8	2.9	3.0	2.6	2.3	1.7	2.4
Snhg20	3.7	3.9	3.4	3.4	3.5	3.4	2.9	3.1
Snx21	4.9	4.5	4.5	4.0	4.5	4.6	4.3	4.1
Srrm4	0.5	0.9	1.5	1.6	-1.0	0.3	2.2	1.2
Srsf5	7.3	7.4	7.6	7.6	7.2	7.2	7.6	7.7
Steap3	3.1	2.7	2.3	2.1	3.0	3.8	3.3	2.7
Tada2b	4.6	4.6	2.4	3.1	1.7	4.0	2.3	1.7
Tcfl5	4.5	4.5	1.9	2.5	0.3	3.5	1.3	0.2
Thap11	5.7	5.5	5.4	5.0	5.5	5.5	5.0	5.1
Thbd	4.2	4.3	4.0	5.3	5.5	5.8	7.1	6.6
Tmbim6	8.3	8.2	7.3	7.8	7.2	8.2	7.9	7.0
Tmem185b	4.5	4.8	5.3	5.2	4.8	4.7	5.3	5.2
Tmem265	1.9	1.7	1.3	1.2	1.7	1.7	0.8	0.4
Tmem69	3.0	3.2	3.6	3.5	2.6	2.6	3.5	3.5
Tra2b	7.0	7.2	7.3	7.6	6.9	7.1	7.6	7.5
Trib1	2.0	2.5	3.4	5.0	2.1	3.3	5.5	5.4
Trim11	5.0	4.9	4.5	4.6	4.7	4.6	4.1	4.5
Trim16	4.8	4.8	5.2	5.8	5.0	4.9	5.1	5.3
Trim6	2.2	2.0	2.5	2.6	2.0	2.2	2.6	2.1
Trmt5	3.6	3.7	4.0	4.1	3.5	3.7	3.7	3.8
Tshz1	3.2	3.3	2.7	2.8	2.6	3.2	2.9	2.4
Tst	1.3	1.2	0.8	0.6	2.3	2.8	2.3	2.0
Ufsp1	3.0	2.9	2.7	2.3	2.9	2.8	2.4	2.6
Uprt	1.6	1.4	1.7	2.0	1.9	2.0	2.4	2.4
Usp49	2.0	3.0	1.7	1.8	2.1	2.0	1.6	1.2
Vgll3	5.3	5.6	5.9	6.0	5.3	5.6	6.1	6.2

Wsb1	6.9	7.0	7.6	7.9	6.9	7.0	8.1	8.2
Wtip	4.9	4.7	1.2	2.6	1.8	4.6	2.4	1.0
Xkr8	3.6	3.5	3.2	3.3	4.4	3.9	3.5	3.6
Zbtb7b	4.1	4.2	3.4	3.7	4.3	4.0	4.1	3.3
Zc2hc1c	1.8	1.1	1.3	0.4	2.2	2.0	1.0	0.9
Zfand5	6.8	6.6	6.9	7.2	6.8	6.9	7.3	7.3
Zfp119a	2.5	2.7	3.4	3.5	2.3	3.0	3.8	3.6
Zfp236	2.8	3.1	1.7	2.8	2.6	2.7	1.9	1.9
Zfp296	3.1	2.9	2.9	2.5	3.8	3.4	2.7	2.7
Zfp30	2.2	1.7	1.9	1.2	2.0	1.6	1.1	1.1
Zfp36	5.1	5.0	6.1	6.1	7.3	4.8	8.2	7.7
Zfp433	3.1	3.1	3.9	3.9	3.2	3.3	4.0	3.6
Zfp668	3.6	3.5	3.0	3.0	3.1	3.4	3.1	2.9
Zfp688	4.6	4.1	4.4	3.7	4.6	4.1	3.6	3.5
Zfp747	2.9	2.7	3.0	3.2	2.4	2.8	3.5	3.3
Zfp947	1.2	1.0	2.0	2.2	0.5	1.6	2.4	1.6
Zfp958	4.7	4.2	4.2	4.3	4.6	4.3	4.0	3.7
Zfp959	3.4	3.2	3.8	4.0	2.6	3.2	3.8	4.0
Zfpm1	4.7	4.5	1.7	3.0	1.8	4.7	2.1	1.2
Zmym1	4.2	4.1	4.3	4.9	4.4	4.6	4.8	4.9
Znrf2	4.6	4.7	1.8	2.6	2.4	4.5	2.7	1.8
Zrsr1	1.3	1.0	0.7	-0.2	1.6	1.2	0.5	0.8

Supplement Table 2: Incongruent Genes used in the heat map.

Gene Symbol	WT static	WT static 2	WT perfused	WT perfused 2	R899X static	R899X static 2	R899X perfused 2	R899X perfused
1500004A13Rik	0.8	1.3	0.2	1.1	0.6	0.9	1.9	1.3
1700030J22Rik	0.4	0.6	-0.3	0.3	-0.3	0.0	0.4	0.7
1810010H24Rik	2.6	2.3	1.4	1.5	0.5	1.6	2.2	1.7
5031425E22Rik	3.2	3.2	3.1	3.1	2.3	2.3	2.9	2.8
6330408A02Rik	2.4	2.4	2.0	2.1	1.5	2.4	2.8	2.6
A630052C17Rik	2.2	2.1	1.7	1.5	0.9	1.6	1.7	1.6
A830082K12Rik	2.0	2.0	2.5	2.5	2.3	2.4	2.1	1.8
A930005H10Rik	2.7	2.6	2.1	1.8	2.0	1.8	2.2	1.5
Abcb9	0.9	0.8	0.2	-0.3	0.4	0.1	0.7	0.3
AC158396.1	3.2	3.3	3.5	3.2	3.3	3.0	2.4	2.7
Acss2	2.0	1.8	1.8	2.0	2.3	2.3	1.8	2.0
Adck4	4.7	4.4	4.7	4.5	5.0	5.0	4.5	4.7
Adm	-0.5	-0.9	0.9	0.8	1.7	-0.2	0.5	1.3
Adrb2	2.4	2.6	2.9	2.6	1.9	1.6	2.8	2.9
Aipl1	1.7	1.7	0.9	0.8	0.9	0.8	1.0	0.7
Alkbh4	4.4	4.3	4.5	4.0	3.9	4.0	3.1	3.2
Ank3	3.0	2.4	1.7	2.8	-1.5	-1.4	-0.2	-0.2
Ap1s2	3.9	4.2	4.5	4.6	3.7	3.7	4.0	3.7
Arc	1.9	1.4	2.4	3.4	0.9	-0.4	3.8	5.2
Arhgef6	2.1	1.7	1.6	2.1	0.9	1.4	2.3	1.9
Armcx5	3.6	3.7	3.4	3.5	3.1	3.6	3.8	4.0
Arsj	1.3	1.4	2.0	2.0	2.4	2.2	1.3	1.9
Asic3	3.8	3.1	3.8	3.5	1.3	1.2	0.4	0.4
Asna1	6.6	6.6	6.1	6.1	5.5	6.0	5.9	5.4
Ass1	3.2	3.6	2.4	2.6	2.5	3.3	3.4	2.4
Atp6v0d1	7.2	7.2	6.4	6.5	5.9	6.6	6.6	6.0
B3galnt1	-0.3	-0.2	-2.0	-2.0	4.7	4.6	4.4	4.7
B4galnt2	1.1	0.7	0.3	0.0	-0.8	0.2	0.0	-0.6

BC023105	4.3	3.7	4.2	3.8	3.8	3.8	4.7	4.3
Bcam	2.9	2.7	2.0	1.3	3.3	3.6	3.5	3.1
Bcas3	5.1	5.0	4.7	4.8	3.5	4.5	4.9	4.6
Bcdin3d	2.8	2.5	2.9	2.3	3.0	2.9	2.5	2.1
Brd3	5.8	5.8	5.2	5.5	5.1	5.7	5.4	5.5
Btg3	4.1	4.2	4.6	4.9	4.6	4.5	4.2	4.2
C630043F03Rik	0.5	1.1	1.2	0.8	1.4	1.2	0.9	0.9
C920006O11Rik	0.7	0.3	-0.3	-0.2	0.4	0.5	0.4	0.2
Cacnb1	0.8	0.4	0.7	0.4	1.1	0.7	-0.1	-0.3
Calr3	1.8	1.6	2.3	1.9	2.1	2.3	1.5	1.8
Ccdc163	1.9	1.7	2.2	2.1	2.6	2.1	1.6	1.9
Cers4	3.4	3.3	3.0	2.6	4.0	4.2	3.7	4.3
Ces2g	4.7	4.9	4.4	4.3	5.8	5.9	5.8	5.9
Cited2	6.0	5.9	6.5	6.0	5.6	6.2	7.1	7.1
Coq10a	5.0	4.8	4.4	4.5	4.7	4.7	4.5	4.7
Csrp1	7.3	7.4	7.2	7.2	6.4	6.5	7.0	6.8
Cyb5d2	2.8	2.8	2.2	2.3	2.4	2.5	2.7	2.6
D130017N08Rik	2.3	2.6	1.9	1.8	1.2	1.4	1.5	1.4
Def6	2.8	2.5	1.8	1.5	3.1	4.0	3.8	3.8
Dlx2	2.3	2.8	3.6	3.9	3.1	3.0	3.2	3.4
E230001N04Rik	1.4	1.7	0.7	0.3	-0.1	1.2	1.0	0.6
Eif3s6-ps4	1.1	0.8	1.2	0.9	-0.1	0.1	0.7	0.8
Eva1b	3.4	3.3	3.3	3.0	4.3	4.0	3.6	3.7
Exoc3l4	4.2	4.2	4.7	4.0	4.4	4.5	3.9	3.7
Fam213a	2.5	2.5	1.6	1.7	3.9	4.1	4.0	4.1
Fam43a	0.7	0.4	-0.2	1.7	-0.2	-0.8	1.3	0.9
Fam53c	6.5	6.2	5.8	6.8	5.3	5.7	6.5	6.2
Fcor	1.6	2.2	2.3	1.7	0.4	0.6	-0.1	0.1
Flot2	7.7	7.5	7.3	7.1	6.9	7.5	7.2	7.2
Fmo5	0.5	0.7	0.0	-0.6	0.5	1.2	1.7	1.4
Fnbp1	3.9	3.8	3.3	3.4	3.4	3.9	3.6	3.5
Fpgt	4.0	4.0	4.5	4.8	4.2	4.3	4.6	4.3
Gadd45a	6.3	6.1	6.6	6.8	6.8	6.2	6.5	5.9
Gadd45b	4.9	5.2	6.0	5.9	5.4	4.7	4.8	5.1
Gale	4.4	4.3	4.7	4.7	4.4	4.0	3.5	3.9
Gata2	3.4	2.9	3.1	3.4	0.9	1.0	1.4	1.4

Gfra4	0.8	1.2	0.4	0.5	1.6	2.1	1.8	1.7
Gimap9	2.2	2.5	2.8	2.4	3.4	3.4	2.6	3.0
Gm10224	1.2	1.2	1.8	1.0	2.0	1.9	1.2	1.4
Gm10335	4.2	4.4	4.5	4.1	4.7	4.5	3.8	4.1
Gm11821	0.6	0.7	0.3	1.0	0.4	0.5	1.0	0.9
Gm11847	-0.6	-0.4	0.4	0.7	0.8	0.9	0.5	0.2
Gm12094	0.4	0.4	0.7	0.5	0.7	0.0	-0.8	-0.5
Gm12185	4.8	4.4	4.7	4.9	4.1	4.7	5.3	5.6
Gm12430	1.4	1.2	1.5	1.5	1.3	1.3	1.7	1.9
Gm12502	0.5	0.1	0.7	0.0	0.5	0.3	-0.6	-0.6
Gm12758	0.2	-0.1	1.0	0.8	-0.6	0.0	-0.6	0.5
Gm12791	0.9	0.7	0.5	0.4	0.2	0.3	0.9	0.6
Gm13147	0.2	0.2	1.0	0.4	0.8	0.6	0.1	0.3
Gm13331	1.9	2.0	2.5	1.9	2.8	2.5	2.0	2.3
Gm13408	2.1	1.8	2.5	2.3	2.9	2.4	2.3	2.3
Gm13743	0.3	0.3	1.0	0.9	0.6	0.5	-0.2	0.3
Gm14434	2.0	2.5	2.4	2.1	1.2	1.5	0.5	0.1
Gm15710	5.8	5.9	6.3	5.5	5.9	5.6	5.0	5.2
Gm18180	1.0	0.8	0.9	1.2	0.1	0.3	0.8	0.8
Gm18853	1.9	2.6	2.2	2.7	2.6	3.4	4.2	4.1
Gm20707	0.8	0.8	1.4	1.3	0.9	1.5	1.4	1.2
Gm28437	8.5	8.7	9.1	9.4	9.4	8.6	9.2	8.7
Gm43110	2.7	2.7	2.9	2.4	2.3	2.2	1.8	1.8
Gm4841	5.6	5.5	5.6	5.5	5.7	5.5	6.1	6.3
Gm5145	0.4	0.4	0.7	0.7	1.0	0.6	-0.2	0.4
Gm5385	-0.3	-0.4	-0.3	-0.1	0.9	1.0	-0.4	0.1
Gm5424	4.8	5.2	4.0	4.1	4.4	5.1	4.8	4.2
Gm6065	1.5	1.5	1.6	1.3	1.7	1.6	0.8	1.1
Gm6576	2.2	2.4	2.7	2.3	2.9	2.6	2.0	2.0
Gm8210	4.4	4.4	4.8	4.2	4.8	4.5	3.7	4.0
Gm8623	0.6	1.3	1.5	0.6	1.6	1.2	0.6	0.7
Gm9795	0.2	0.2	-0.6	-1.3	0.7	1.1	1.6	0.5
Gm9826	0.7	0.5	1.3	1.1	2.9	2.9	2.6	2.5
Gpr176	-1.0	-1.2	-1.8	-2.0	3.5	4.5	4.7	4.7

Gpt	2.2	1.8	1.4	1.2	1.3	1.5	2.0	1.3
Gstcd	2.4	2.9	2.4	2.5	3.2	3.2	2.3	2.3
Hacl1	3.9	3.8	3.4	3.2	3.1	3.5	3.6	3.4
Hcn2	1.5	1.7	-1.2	0.2	-0.9	0.5	0.2	-1.4
Hist1h1b	0.6	0.5	1.6	1.7	1.7	0.3	1.1	0.5
Hist1h2ad	4.6	5.0	5.1	5.0	3.5	3.3	2.7	2.8
Hist1h2ap	6.9	7.3	7.5	7.3	7.7	7.2	6.6	6.8
Hmgb1-ps1	1.6	1.7	2.2	1.0	1.7	1.7	0.5	1.0
Hpdl	0.8	0.8	1.5	1.3	1.4	0.8	-0.4	0.5
Itga7	1.3	1.2	1.3	1.6	3.2	3.2	2.9	2.8
Katnbl1	5.7	5.7	6.1	6.2	6.5	6.1	6.3	6.4
Kazn	2.0	2.2	1.5	1.4	3.0	3.8	4.2	3.5
Krt19	7.0	7.1	7.1	6.9	6.9	6.6	6.2	6.1
Lama5	3.4	3.8	2.6	2.5	3.5	4.6	4.8	3.5
Lims2	4.2	3.9	3.9	3.0	1.5	1.4	1.8	1.7
Lppr2	2.4	2.5	1.9	1.7	1.9	2.4	2.6	2.1
Lyrm4	3.1	3.4	2.2	2.7	3.4	3.7	3.5	3.4
Lzts2	6.4	6.1	5.8	5.8	6.0	6.5	6.2	6.1
Map9	0.4	1.3	0.1	0.4	-1.3	-0.6	0.7	0.3
Mdm2	7.8	7.5	7.4	7.8	7.2	7.2	7.7	7.7
Mettl23	3.3	3.4	3.0	2.8	3.1	3.5	3.4	3.2
Mfsd2a	-0.9	-0.1	1.4	1.5	1.5	-0.6	0.7	0.9
Mir17hg	0.2	0.5	0.5	0.4	-1.0	-0.1	0.9	1.0
Mme	2.8	2.6	2.2	3.0	1.9	1.9	3.0	2.7
Mpv17l	1.7	1.5	2.1	2.3	2.1	1.5	2.0	1.6
Mtfp1	1.4	1.3	1.3	1.2	0.4	0.4	0.0	-0.2
Mum1l1	3.9	4.0	3.8	4.4	3.4	3.8	4.2	4.5
Mzf1	0.9	0.9	0.1	0.3	0.3	0.3	0.3	0.1
Nat14	1.6	1.3	1.4	1.1	2.1	2.0	1.4	1.1
Neurl4	2.2	2.6	1.2	1.6	1.6	2.1	2.3	1.3
Noxo1	1.8	1.2	0.7	0.8	-0.3	0.7	0.7	1.3
Nqo1	7.2	7.2	7.8	7.5	8.9	8.4	8.2	8.7
Nr1h4	3.8	3.4	3.8	3.2	3.1	3.4	3.9	3.8
Nr4a2	-0.6	0.0	0.8	1.5	-1.2	-0.9	4.2	3.7
Nrg1	1.3	1.5	1.6	1.1	2.2	2.0	2.6	2.5

Nudt17	1.6	1.8	1.8	1.2	2.9	2.9	2.1	2.2
P2ry2	3.4	3.1	3.0	3.6	3.1	3.0	3.7	3.6
Paqr7	3.6	3.9	3.0	3.0	3.3	3.9	3.4	3.5
Pcdhgc5	1.2	1.6	0.2	0.0	0.5	1.1	0.9	1.3
Pcsk4	2.3	1.6	2.6	2.2	3.1	2.8	2.3	2.2
Phtf1os	1.6	1.5	0.4	-0.2	0.6	0.9	1.1	1.0
Plekha7	2.2	2.0	0.3	1.0	1.8	2.6	2.8	2.7
Ppm1m	4.6	4.6	4.1	4.2	3.8	4.3	4.2	4.0
Ppp1r10	5.0	4.9	4.4	4.9	4.4	4.6	5.7	5.6
Prdm16	2.7	2.6	2.2	2.4	2.3	2.8	2.9	2.7
Ptms	4.4	4.7	4.0	3.9	3.8	3.9	4.0	3.4
Ralgds	1.0	1.1	-0.2	0.3	1.5	2.0	2.3	2.3
Rasa4	2.0	1.3	1.4	1.7	1.6	2.0	1.0	1.2
Rbm12b1	2.6	2.4	2.3	2.8	2.6	2.6	3.0	3.1
Rbm5	5.9	5.8	5.8	6.0	5.7	6.0	6.5	6.3
Rgl1	0.6	0.1	0.7	0.1	1.1	0.8	0.3	0.5
Ribc1	1.7	1.0	1.5	1.1	1.8	1.6	1.3	1.1
Rnf130	3.4	3.3	2.6	2.1	3.7	4.6	4.5	4.2
Rorc	2.0	1.7	0.9	1.1	1.4	1.9	2.3	1.2
Rpgrip1l	1.4	2.2	1.5	2.0	0.1	1.1	2.2	2.0
Rpl28-ps3	1.2	1.6	1.9	1.5	1.6	1.4	0.7	1.1
Rpl31-ps10	0.9	1.2	1.3	0.5	1.7	1.5	0.9	1.0
Rpl31-ps16	1.2	1.3	1.4	0.7	1.6	1.2	0.4	0.8
Rragd	3.1	3.1	2.1	2.5	2.5	2.7	3.2	3.1
Rsad2	8.6	7.8	7.8	8.4	7.3	7.0	7.8	8.0
Rsbn1	3.3	3.1	3.6	3.9	3.7	3.7	4.0	3.4
Sass6	3.4	3.0	3.1	3.6	2.2	2.5	3.2	3.0
Scn1b	1.7	1.5	0.2	0.1	0.7	1.1	0.5	1.0
Sco2	2.6	2.7	3.5	3.2	3.1	2.5	2.9	2.9
Sdsl	2.5	2.6	1.9	1.7	0.5	0.4	0.8	0.0
Sema3b	3.6	3.5	2.9	2.6	1.0	2.0	2.3	1.3
Serpina3g	0.5	0.0	-1.8	-1.6	5.0	5.3	5.7	4.9
Serpina3n	1.8	1.6	1.0	0.7	4.7	4.9	5.2	4.6
Slc25a42	4.4	4.0	3.3	3.5	3.2	3.4	3.5	3.1
Slc37a4	4.1	3.5	3.8	3.5	4.4	4.1	3.5	3.5
Slc41a3	4.3	4.1	3.9	3.7	3.4	3.3	3.5	3.3
Slc44a3	0.0	-0.5	-1.6	-1.2	2.9	2.5	2.5	2.5
Smim19	5.6	5.7	4.9	5.0	4.9	5.1	5.0	4.9

Snai2	5.9	5.8	6.3	6.5	6.3	6.1	6.2	6.0
Snhg17	4.0	4.1	5.1	4.6	5.0	4.0	5.0	4.2
Sord	5.1	4.7	4.2	4.3	5.1	4.9	5.0	5.0
Speer3	-1.2	-2.0	0.2	-0.1	0.7	1.3	1.1	1.4
St3gal3	2.2	2.4	2.0	1.7	2.0	2.3	2.0	2.1
Syt12	3.4	3.6	3.7	3.6	2.5	2.5	2.0	2.1
Sytl1	3.2	3.2	2.5	2.0	2.6	3.0	2.9	2.7
Tab1	4.9	4.3	4.6	4.7	4.7	4.8	4.3	4.0
Taf1c	3.7	3.8	3.8	3.9	3.6	3.6	4.0	4.1
Taf5	4.4	4.0	4.1	4.4	4.3	4.1	3.8	3.9
Tec	0.0	0.3	-0.7	-0.8	1.2	1.8	1.5	2.0
Tmem121	3.6	3.4	3.6	2.9	4.3	3.9	3.1	3.3
Tmem170	1.0	0.8	0.4	1.0	0.5	0.3	1.5	1.2
Tmem25	1.0	0.3	0.4	0.1	0.2	0.9	1.9	1.7
Tmem86a	1.3	1.3	0.4	0.4	0.8	0.3	0.2	0.8
Tnip1	5.2	4.9	4.4	4.7	4.5	4.9	5.1	4.5
Tprn	4.2	3.6	4.0	3.7	4.1	3.9	3.5	3.5
Trim12a	5.1	4.9	5.0	5.1	4.3	4.5	4.8	4.9
Trim34b	-1.4	-1.3	-1.1	-0.6	2.0	2.1	1.7	1.8
Trim56	3.3	4.0	3.3	4.0	2.8	2.6	3.7	3.3
Trim7	1.5	1.6	0.9	0.9	-0.1	0.6	0.6	0.6
Ttc12	0.0	0.6	0.3	0.0	-0.5	0.1	0.6	0.7
Ttpa	1.7	1.7	2.3	2.2	0.3	0.8	0.8	0.4
Tuft1	6.6	6.8	6.8	6.6	5.7	5.9	6.2	6.3
Ube2e2	6.0	5.9	5.4	5.2	4.9	5.4	5.1	5.1
Usp2	1.7	2.2	2.3	2.0	1.7	1.5	3.5	2.8
Vsig10l	0.8	0.8	0.6	0.4	1.1	1.3	1.8	1.5
Wars2	3.9	3.9	4.3	4.4	4.3	4.2	4.1	4.1
Wsb2	8.0	8.1	7.8	7.7	6.8	7.3	7.4	7.6
Ypel2	0.6	0.3	-0.8	-0.9	-0.9	0.9	1.6	0.5
Zcwpw1	1.5	1.1	0.3	0.4	-0.6	0.6	0.8	0.8
Zfp362	2.9	3.2	2.0	1.9	1.5	3.0	2.3	2.1
Zfp472	3.1	3.2	3.7	3.6	3.0	3.3	3.3	3.1
Zfp566	3.5	3.2	3.9	3.7	3.9	3.5	3.8	3.7
Zfp763	3.1	3.0	2.4	3.3	2.9	3.3	2.2	2.1
Zfp772	3.2	3.0	3.0	3.3	3.1	2.8	2.6	2.4

Zfp775	4.1	3.7	3.9	3.7	4.1	4.0	3.7	3.3
Zfp81	2.9	2.8	3.6	3.6	4.0	3.2	3.4	3.4
